# Molecular Subtypes Based on the Stemness Index Predict Prognosis in Glioma Patients

**DOI:** 10.3389/fgene.2021.616507

**Published:** 2021-03-01

**Authors:** Jun Tan, Hecheng Zhu, Guihua Tang, Hongwei Liu, Siyi Wanggou, Yudong Cao, Zhaoqi Xin, Quanwei Zhou, Chaohong Zhan, Zhaoping Wu, Youwei Guo, Zhipeng Jiang, Ming Zhao, Caiping Ren, Xingjun Jiang, Wen Yin

**Affiliations:** ^1^Department of Neurosurgery, Xiangya Hospital, Central South University, Changsha, China; ^2^Changsha Kexin Cancer Hospital, Changsha, China; ^3^Department of Clinical Laboratory, Hunan Provincial People’s Hospital (First Affiliated Hospital of Hunan Normal University), Changsha, China; ^4^Hunan International Scientific and Technological Cooperation Base of Brain Tumor Research, Xiangya Hospital, Central South University, Changsha, China; ^5^Key Laboratory for Carcinogenesis of Chinese Ministry of Health, School of Basic Medical Science, Cancer Research Institute, Central South University, Changsha, China

**Keywords:** molecular subtypes, stemness index, glioma, prognostic signature, immune infiltration

## Abstract

Glioma is the common histological subtype of malignancy in the central nervous system, with high morbidity and mortality. Glioma cancer stem cells (CSCs) play essential roles in tumor recurrence and treatment resistance. Thus, exploring the stem cell-related genes and subtypes in glioma is important. In this study, we collected the RNA-sequencing (RNA-seq) data and clinical information of glioma patients from The Cancer Genome Atlas (TCGA) and Chinese Glioma Genome Atlas (CGGA) databases. With the differentially expressed genes (DEGs) and weighted gene correlation network analysis (WGCNA), we identified 86 mRNA expression-based stemness index (mRNAsi)-related genes in 583 samples from TCGA RNA-seq dataset. Furthermore, these samples from TCGA database could be divided into two significantly different subtypes with different prognoses based on the mRNAsi corresponding gene, which could also be validated in the CGGA database. The clinical characteristics and immune cell infiltrate distribution of the two stemness subtypes are different. Then, functional enrichment analyses were performed to identify the different gene ontology (GO) terms and pathways in the two different subtypes. Moreover, we constructed a stemness subtype-related risk score model and nomogram to predict the prognosis of glioma patients. Finally, we selected one gene (ETV2) from the risk score model for experimental validation. The results showed that ETV2 can contribute to the invasion, migration, and epithelial-mesenchymal transition (EMT) process of glioma. In conclusion, we identified two distinct molecular subtypes and potential therapeutic targets of glioma, which could provide new insights for the development of precision diagnosis and prognostic prediction for glioma patients.

## Introduction

Gliomas are the most common primary malignant tumor of the central nervous system (Ostrom et al., 2014). Therapeutic strategies, including surgery, chemotherapy, and radiotherapy, have been widely applied, but the overall outcome of glioma patients is still unsatisfactory (Bush et al., 2017; Nabors et al., 2017). The heterogeneity of glioma is an account of the poor prognosis (Klughammer et al., 2018). Thus, exploring the molecular mechanism of glioma may facilitate the identification of prognostic biomarkers and potential targets for the treatment of glioma.

Cancer stem cells (CSCs) are a subset of cancer cells with characteristics such as the ability to self-renew long-term, differentiate into defined progenies, and sustain tumor growth (Vlashi and Pajonk, 2015; Clarke, 2019). CSCs contribute to glioma recurrence, radioresistance, and chemoresistance through multiple molecular mechanisms (Prieto-Vila et al., 2017; Schulz et al., 2019). Therefore, a further understanding of the biological behavior of glioma stem cells may facilitate to changes in the treatment dilemma of glioma. Recently, the mRNA expression-based stemness index (mRNAsi), which represents the transcriptomic stemness expression, has been applied to assess CSC characteristics (Malta et al., 2018). In some cancers, such as bladder, lung, breast, or endometrial carcinoma, it has been reported that mRNAsi is a credible marker and is associated with tumor stage (Pan et al., 2019; Liu et al., 2020; Pei et al., 2020). However, its roles in glioma are rare known.

In this study, we collected the RNA-sequencing (RNA-seq) data and clinical information of glioma patients from The Cancer Genome Atlas (TCGA) and Chinese Glioma Genome Atlas (CGGA) databases. We found that glioma patients could be divided into two significant stemness subtypes (S1 and S2 groups) based on mRNAsi-related genes. We identified that the clinical characteristics, such as age, IDH status, and WHO grades, were different in the S1 and S2 groups. The tumor microenvironments in the two groups were also different. Furthermore, based on differentially expressed genes (DEGs) between the two stemness subtypes, a prognostic prediction model was constructed and could effectively divide patients into different prognoses in both TCGA and CGGA datasets. Finally, we selected one gene (ETV2) from the risk score model for further experimental validation. The results showed that ETV2 was more highly expressed in glioma and contributed to the invasion, migration, and epithelial-mesenchymal transition (EMT) process of glioma. Thus, our study provides novel molecular subtypes based on the stemness index to predict prognosis in glioma patients who may promote the clinical diagnosis and treatment of glioma.

## Materials and Methods

### Data Preparation

**Figure 1:**
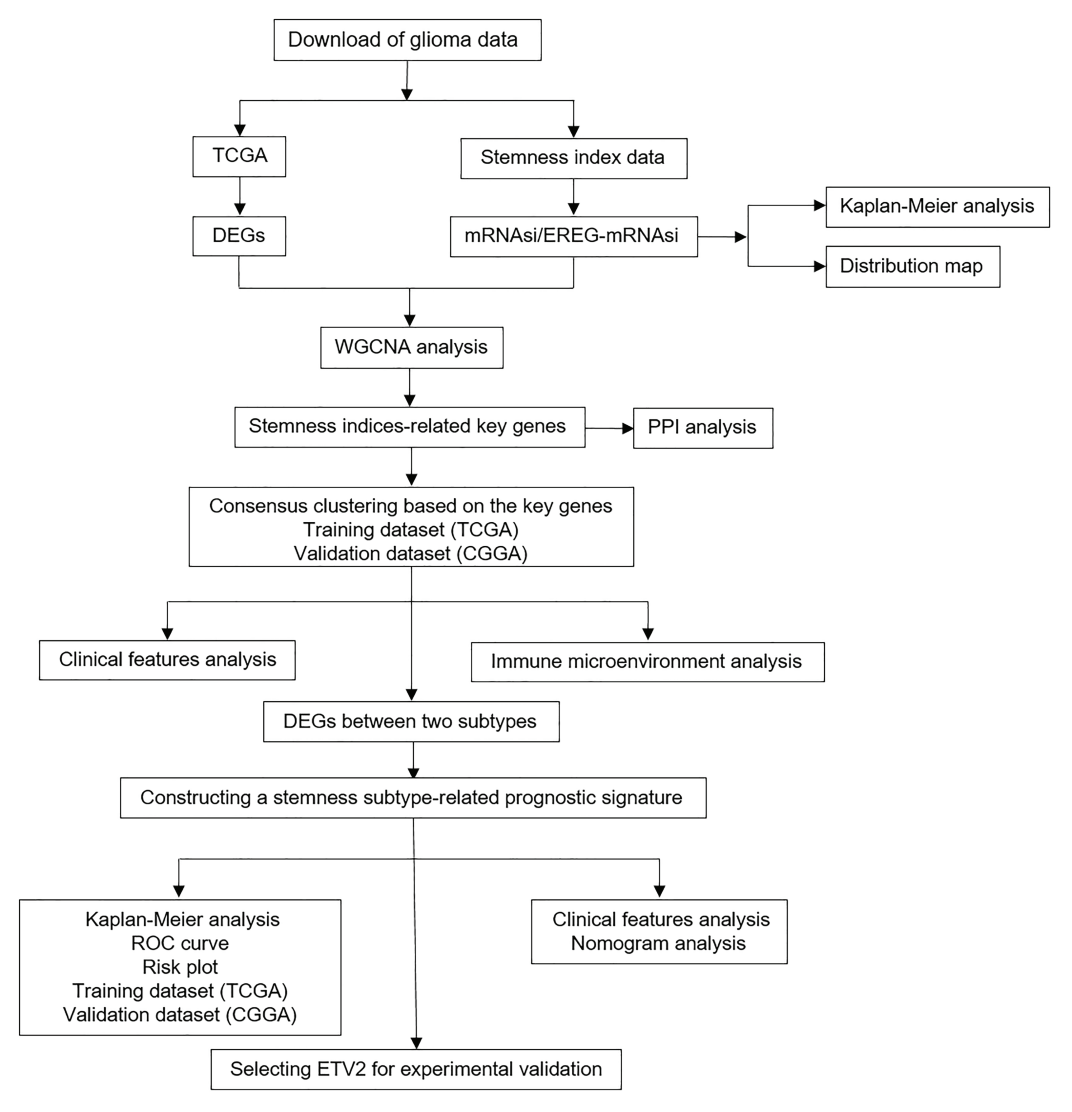
Flowchart presenting the process of analysis.

Figure 1 shows the workflow of data analysis. The RNA-seq data and corresponding clinical data of glioma (lower-grade glioma, LGGs and glioblastoma, GBM) and normal samples in TCGA database were downloaded from the UCSC Xena database^1^ (dataset ID: TCGA.GBMLGG.sampleMap/HiSeqV2). In addition, another glioma dataset (RNA-seq and clinical data) was downloaded from the CGGA^2^ (dataset ID: mRNAseq_693). The mRNAsi indices of glioma in TCGA were obtained from a previous study (Malta et al., 2018). The tumor purity of glioma is calculated based on the Estimation of STromal and Immune cells in MAlignant Tumors using Expression data (ESTIMATE) algorithm, which can predict the level of infiltrating stromal and immune cells and then infer the tumor purity of tumor tissue (Yoshihara et al., 2013).

### Differentially Expressed Genes Analysis

The “limma” R package was utilized to perform the DEG analysis between glioma and normal samples in TCGA database. |Log_2_(Fold change)| > 1 and false discovery rate (FDR) < 0.05 were considered as the cutoff criteria. The volcano plot was drawn to show the DEGs.

### Weighted Gene Correlation Network Analysis

The selected DEGs were used in weighted gene correlation network analysis (WGCNA) by the WGCNA R package (Langfelder and Horvath, 2008). After filtering the outliers in RNA-seq data, a Pearson correlation matrix was constructed for paired genes. Then, we established a weighted adjacency matrix by the power function amn = |cmn|*β*, as a previous study described (Yu et al., 2012). A proper *β* value was selected to increase the matrix similarity and establish a co-expression network. Next, the adjacency matrix was converted into a topological overlap matrix (TOM) to measure gene connectivity in the network. Based on TOM-based dissimilarity measurements, average linkage hierarchical clustering was performed with a minimum gene dendrogram size over 30. Finally, their dissimilarity was calculated, and module dendrograms were constructed for further analysis.

Gene significance (GS), representing the correlation between genes and sample traits, was calculated for each module. In the principal component analysis of each module, module eigengenes (MEs) were considered as the first principal component of a clustered module representing the gene expression profiles. Module membership (MM) was defined as the correlation between the module genes and gene expression profiles. In our study, mRNAsi and epigenetically regulated mRNAsi (EGER-mRNAsi) were the selected clinical phenotypes for further analysis. When GS and MM were highly correlated (more than 0.7), the module’s own genes were considered as significantly correlated with clinical traits.

### Consensus Clustering

After finding the genes highly correlated with mRNAsi and EGER-mRNAsi, we performed consensus clustering to divide patients into different stemness subtypes based on these genes. The R package “ConsensusClusterPlus” was adopted to perform the consensus clustering (Wilkerson and Hayes, 2010). The optimal number of subgroups was determined by the cumulative distribution function (CDF) and consensus matrices. In addition, the CGGA dataset was used to validate this clustering.

### Protein-Protein Interaction Analysis

To explore the protein-protein interaction (PPI) network of the selected genes, they were imported into the STRING database,^3^ which is a web tool used to explore the interactions between multiple proteins.

### Comparison of Stemness Subtypes

To investigate the difference between the two stemness subtypes in TCGA and CGGA datasets, we used two-sample *t*-tests to compare different clinical variables such as age, IDH status, and WHO grades in the subtypes. Moreover, stratified survival analysis in different WHO grades was also used to evaluate the prognostic predictive value of the stemness subtypes.

### Profiling of Immune Infiltrates in the Two Stemness Subtypes

Becht et al. (2016) designed the microenvironment cell populations-counter (MCP-counter) method, which can robustly quantify the absolute abundance of eight immune (T cells, CD8 T cells, cytotoxic lymphocytes, NK cells, B lineage, monocytic lineage, myeloid dendritic cells, and neutrophils) and two stromal cell (endothelial cells and fibroblasts) populations in heterogeneous tissues from transcriptomic data. The MCPs were analyzed by the “MCPcounter” R package. Based on this package, the proportions of eight tumor-infiltrating immune cells (T cells, CD8 T cells, cytotoxic lymphocytes, NK cells, B lineage, monocytic lineage, myeloid dendritic cells, and neutrophils) and two non-immune cells (endothelial cells and fibroblasts) were calculated based on the normalized RNA-seq data in TCGA and CGGA databases.

### Functional Enrichment Analysis

First, DEG analysis between two stemness subtypes was performed. Then, Gene Ontology (GO) and Kyoto Encyclopedia of Genes and Genomes (KEGG) analyses were performed with the Database for Annotation, Visualization and Integrated Discovery (DAVID) to explore the different mechanisms and pathways between the two subtypes (Huang et al., 2009a,b).

Gene set enrichment analysis (GSEA) was performed between the two stemness subtypes with GSEA software. The reference gene set (c2.cp.kegg.v7.1.symbols.gmt) was acquired from the MSigDB database.^4^ Only enriched KEGG pathways with a *p* < 0.05 and FDR < 0.25 were considered statistically significant.

### Construction and Validation of a Stemness Subtype-Related Prognostic Signature

First, DEG analysis between two stemness subtypes was performed in TCGA dataset as previously described. Then, univariate Cox hazard analysis was performed with *p* < 0.05 as a threshold parameter for the DEGs between the two stemness subtypes. By applying the “glmnet” R package, the least absolute shrinkage and selection operator (LASSO) algorithm was used to construct the stemness subtype-related prognostic signature (Goeman, 2010). This signature was created utilizing Cox regression coefficients to multiply the expression values of the select genes. According to the median of risk score, the patients were divided into high and low-risk groups. In addition, the time-dependent ROC curve and Kaplan-Meier survival curve analyses were used to evaluate the accuracy of the signature. The signature was also validated with CGGA dataset.

### Nomogram Construction

To evaluate the predictive value of the prognostic signature, univariate and multivariate Cox regression analyses were performed together with the clinical information (grade, age, sex, IDH1, and 1p/19q status). Then, a nomogram was constructed to predict the survival probability by using the “rms” R package (Yin et al., 2020). Calibrations were used to evaluate the accuracy of the nomogram.

### Cell Culture, Real-Time Quantitative PCR

HEB, SHG44, and A172 glioma cells were provided by Xiangya Medical School of Central South University, Changsha, China. HEB, A172, and SHG44 cells were cultured in DMEM high glucose medium (Gibco/Thermo Fisher Scientific, Inc.) with 10% fetal bovine serum at 37°C, 5%CO_2_. SiRNAs were transfected with Lipofectamine 2000 (Thermo Fisher Scientific) 48 h before analysis. The siRNAs against the ETV2 gene were synthesized by RiboBio Corporation (Product number: siG000002116A-1-5, Guangzhou, China). Total RNA from HEB, SHG44, and A172 cells was extracted by the Trizol lysis method. cDNA synthesis was performed according to the Thermo Scientific RevertAid First Strand cDNA Synthesis Kit (Thermo Scientific, Waltham, MA). The RNA levels of ETV2 were detected by using real-time quantitative PCR (qRT-PCR) according to the manufacturer’s protocol. The expression of ETV2 and GAPDH was analyzed by the 2^-*Δ*ΔCt^ method. The primers were obtained from Sangon (Shanghai, China) and the sequences were designed as follows: for ETV2, the forward primer was 5'-CTGGAAAGGTACAAGCTCATCC-3' and the reverse primer was 5'-AACTTCTGGGTGCAGTAACGC-3'. For GAPDH, the forward primer was 5'-CATTGACCTCAACTACATGGTT-3' and the reverse primer was 5'-CCATTGATGACAAGCTTCCC-3'.

### Wound Healing and Transwell assay

Wound healing and transwell assays were performed as previously described (Jia et al., 2018).

### Western Blots

A172 and SHG44 cells were lysed with RIPA buffer for half an hour at 4°C. The supernatant was collected and boiled at 95°C for 5–8 min in SDS loading buffer. Then, they were subjected to electrophoresis in 10% SDS-polyacrylamide gels and transferred to the polyvinylidene difluoride membranes. The membranes were blocked with 5% non-fat milk in phosphate-buffered saline (PBS) for 1 h before being incubated with the primary antibody at 4°C overnight. The primary antibodies for western blotting used in this study were GAPDH, ETV2 (ab181847, Abcam), N-cadherin (22018-1-AP, proteintech), and vimentin (10366-1-AP, Proteintech). Then the cells were washed three to four times with 0.1% PBST and incubated with horseradish peroxidase (HRP)-conjugated secondary antibody (1:10,000) for 1 h at room temperature. The membranes were washed in 0.1% PBST four times before exposure. Chemiluminescent HRP substrate was purchased from Millipore (Catalog: WBKLS0500). Images were acquired in a Bio-Rad Universal Hood II machine with Image Lab software.

### Immunohistochemistry

These experiments were approved by the Human Ethics Committee of Xiangya Hospital, and informed consent was obtained from all patients. Based on polyformalin-fixed and paraffin-embedded tissues obtained from GBM patients, immunohistochemistry analysis was conducted. The tissue sections were first deparaffinized and hydrated for antigen retrieval. They were then incubated with 0.3% hydrogen peroxide for 10 min at room temperature and washed twice with PBS. After blocking with 5% goat serum for 10 min, the sections were washed with PBS and incubated overnight with a primary antibody against the ETV2 antibody (1:100, Abcam, ab181847) at 4°C. A horseradish peroxidase-labeled secondary antibody (1:400, Abcam, United States) was added dropwise to the sections, and incubation was carried out at 37°C for 30 min. After washing with PBS, the sections were developed using a DAB substrate kit (Sangon Biotech, Shanghai, China) and counterstained with hematoxylin (Sangon Biotech).

### Statistical Analysis

The R software (version 3.5.1) and GraphPad Prism (version 7.0.0) were used in statistical analyses, and a *p* < 0.05 was considered significant. The log-rank test was conducted in the Kaplan-Meier survival analysis. The Student’s *t*-test was used to compare two groups comparing.

## Results

### The mRNAsi and Clinical Characteristics in Glioma

**Figure 2:**
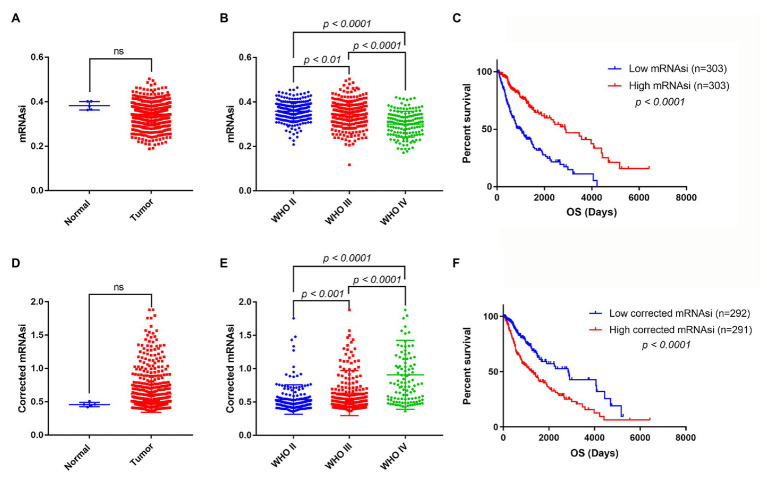
The mRNA expression-based stemness index (mRNAsi), corrected mRNAsi and clinical characteristics in glioma. **(A)** The expression level of mRNAsi in normal (five samples) and tumor (606 samples) tissues. **(B)** Expression level of mRNAsi in gliomas of different WHO grades. **(C)** Kaplan-Meier survival analysis of the relationship between mRNAsi and overall survival (OS). **(D)** The expression level of corrected mRNAsi (mRNAsi/tumor purity) in normal (five samples) and tumor (583 samples) tissues (23 samples cannot calculate the tumor purity). **(E)** The expression level of corrected mRNAsi in glioma of different WHO grades. **(F)** Kaplan-Meier survival analysis of the relationship between corrected mRNAsi and OS.

mRNA expression-based stemness index is a useful indicator that can estimate the number of CSCs by assessing the similarity and heterogeneity between the tumor cells and stem cells. To explore mRNAsi in glioma, the mRNAsi between normal and glioma samples was calculated using TCGA dataset. However, there was no significant difference of mRNAsi between normal and tumor samples (Figure 2A). Next, the correlation between mRNAsi and WHO grades was analyzed. The results showed that different stages of glioma had significantly different mRNAsi values (Figure 2B). Furthermore, the predictive potency of mRNAsi for patient survival prediction was also examined. The Kaplan-Meier analysis showed that higher mRNAsi was significantly associated with a better prognosis of glioma patients (Figure 2C).

Because tumor tissues consist of not only tumor cells but also stromal and immune cells, it reminds us that tumor purity is an important factor interfering with the evaluation of mRNAsi in clinical characteristics. To exclude the potential confounding effect of tumor purity on the analysis, the corrected mRNAsi (mRNAsi/tumor purity) was calculated as previously reported (Pan et al., 2019). We reanalyzed the corrected mRNAsi in normal and glioma samples, but still found no significant difference between them (Figure 2D). However, we found that the corrected mRNAsi values were positively correlated with WHO grades of glioma (Figure 2E). Moreover, we found that patients with higher corrected mRNAsi values had poor prognosis (Figure 2F).

### Screening of DEGs and Identification of Key Genes-Related to mRNAsi

**Figure 3:**
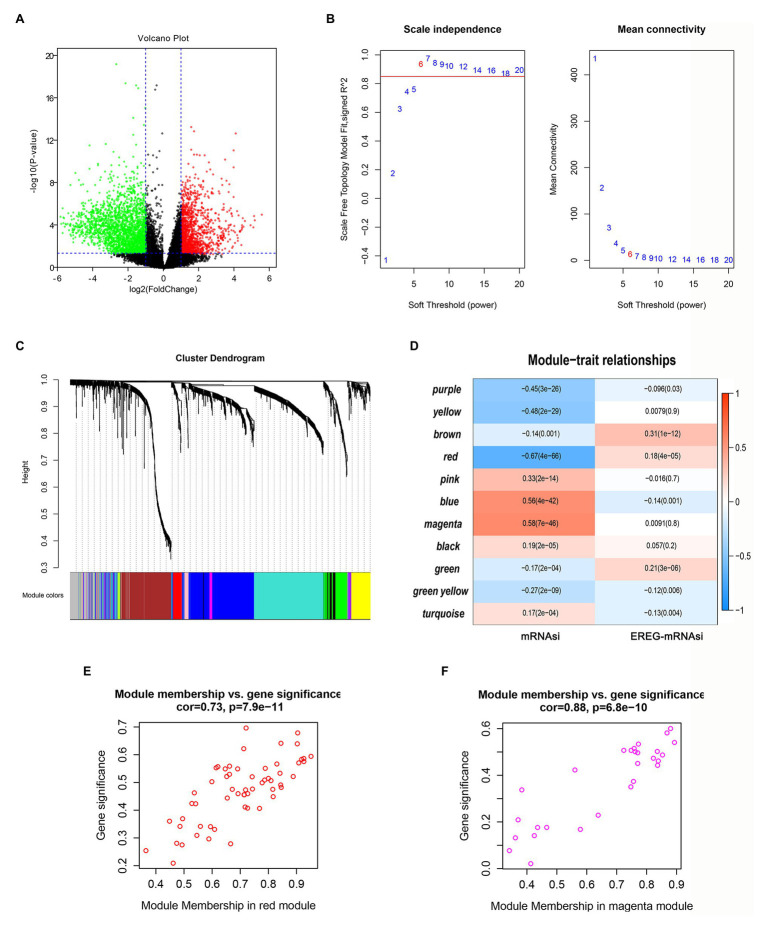
Screening of critical genes related by mRNAsi. **(A)** Volcano plot of differentially expressed genes (DEGs); red represents upregulated genes, and green indicates downregulated genes. **(B,C)** Weighted gene correlation network analysis (WGCNA) of DEGs. Different colors represent different modules. **(D)** Correlation analysis of the modules and clinical traits with mRNAsi or EREG-mRNAsi. Scatter plot analysis of modules in the red **(E)** and magenta **(F)** modules.

First, DEG analysis was performed to compare glioma and normal samples. From this analysis, 3,918 DEGs were screened, of which 1,409 were upregulated, and 2,509 were downregulated (Figure 3A).

To identify the key genes related to mRNAsi, WGCNA analysis was applied based on the selected DEGs. In total, we identified 11 modules (Figures 3B,C), among which the blue and magenta modules exhibited positive correlations with mRNAsi and the red module showed a negative correlation with mRNAsi, with the correlations greater than 0.5 or less than −0.5 (Figure 3D). After calculating the correlations between GS and MM of the three modules, we found that the correlations between GS and MM in the red and magenta modules were highly correlated (more than 0.7; Figures 3E,F). Therefore, the two modules were chosen for further analyses.

### Molecular Subtypes of Glioma Based on mRNAsi-Related Genes

**Figure 4:**
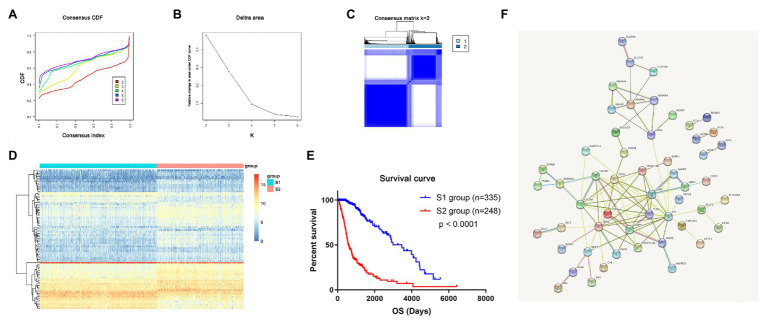
The mRNAsi-related genes could classify glioma into two groups by consensus clustering of TCGA dataset. **(A)** Cumulative distribution function (CDF) for *k* = 2 to *k* = 6. **(B)** Relative change in area under the CDF curve according to different *k* values. **(C)** Consensus clustering matrix of 583 samples from TCGA dataset for *k* = 2. **(D)** Heatmap of two clusters defined by the expression of mRNAsi genes. **(E)** Survival analysis of patients in the S1 group and S2 group in TCGA cohort. **(F)** Protein-protein interaction (PPI) network of the mRNAsi-related genes.

In total, the expression profiling of 86 mRNAsi-related genes in 583 samples from TCGA RNA-seq dataset was obtained. The clinical features of these patients are shown in Table 1. Consensus clustering was performed in the 584 samples, and patients could be divided into two significantly different subtypes (S1 and S2 groups; Figures 4A–C). The heatmap of the two subtypes is also been shown in Figure 4D. Compared with patients in the S1 group, glioma patients in the S2 group showed a shorter overall survival (OS; *p* < 0.0001; Figure 4E). The PPI network analysis showed that most of the mRNAsi-related genes were closely correlated and centered on the stemness-related molecules, such as CD44, CD68, IL6, and CXCR4 (Wang et al., 2019; Ma et al., 2020; Osman et al., 2020).

**Table 1:** Clinical characteristics of 583 glioma patients from The Cancer Genome Atlas (TCGA) cohort included in this study.

Variables	Number (%)
Vital status	
Alive	370 (63.46%)
Dead	213 (36.54%)
Age	
<60	460 (78.90%)
≥60	123 (21.10%)
Sex	
Female	248 (42.54%)
Male	335 (57.46%)
Tumor grade	
WHO II	224 (38.42%)
WHO III	238 (40.82%)
WHO IV	121 (20.76%)
Molecular subtypes	
IDH mutant and 1p/19q codeletion (LGG)	145 (24.87%)
IDH mutant and 1p/19q non-codel (LGG) IDH wildtype (LGG) IDH mutant (GBM) IDH wildtype (GBM)	234 (40.14%) 80 (13.73%) 7 (1.20%) 101 (17.32%)
Others	16 (2.74%)

LGG, lower-grade glioma; GBM, glioblastoma.

**Figure 5:**
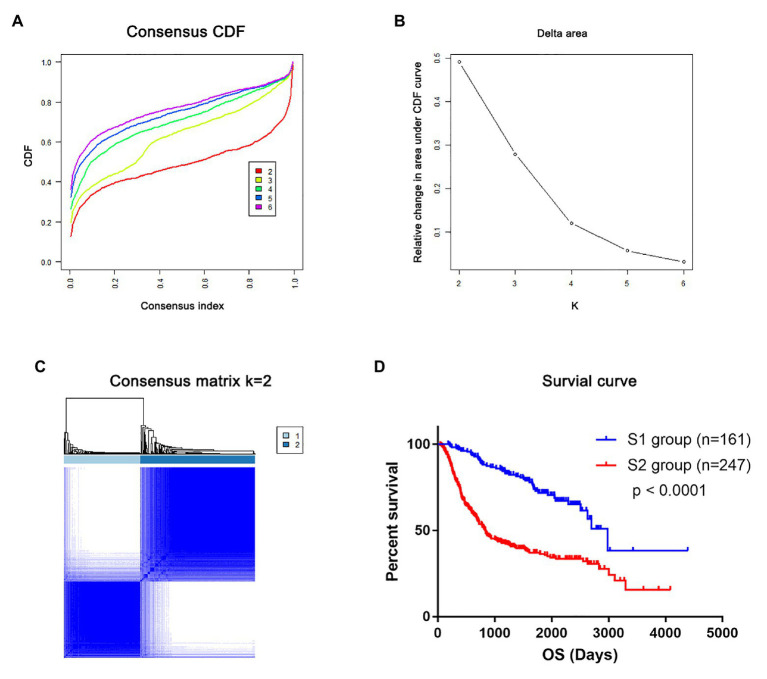
The mRNAsi-related genes could classify glioma into two groups by consensus clustering of the Chinese Glioma Genome Atlas (CGGA) dataset. **(A)** CDF for *k* = 2 to *k* = 6. **(B)** Relative change in area under the CDF curve according to different *k* values. **(C)** Consensus clustering matrix of 408 samples from TCGA dataset for *k* = 2. **(D)** Survival analysis of patients in the S1 group and S2 group in TCGA cohort.

To validate that mRNAsi-related genes could predict prognostic subtypes, the same method with consensus clustering was applied to the CGGA dataset. Interestingly, the patients also could be divide into two distinct subtypes (S1 and S2 groups; Figures 5A–C). Patients in the S2 group also had significantly worse OS (*p* < 0.0001; Figure 5D).

### Clinical Characteristics of Two Stemness Subtypes in the Glioma

**Figure 6:**
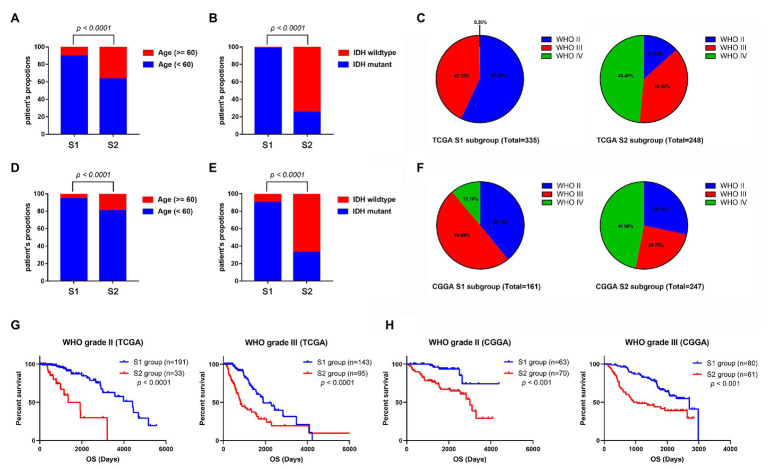
Comparison of the clinical characteristics between the two subtypes using TCGA and CGGA datasets. Histograms to showing that the S2 group of TCGA dataset had significantly more elderly patients **(A)** and more patients with IDH wild-type **(B)**. **(C)** Pie charts show that the S2 group of TCGA datasets has more patients with WHO grade IV glioma and fewer patients with WHO II glioma than the S1 group (*p* < 0.001). Histograms to show that the S2 group of CGGA dataset has significantly more elder patients **(D)** and more patients with IDH wild-type **(E)**. **(F)** Pie charts show that the S2 group of TCGA datasets has more patients with WHO grade IV glioma and fewer patients with WHO II glioma than the S1 group (*p* < 0.001). **(G)** Kaplan-Meier survival curve of S1 and S2 group in different WHO grades (TCGA dataset). **(H)** Kaplan-Meier survival curve of S1 and S2 group in different WHO grades (TCGA dataset).

To identify the clinical characteristics of the two stemness subtypes, we compared the age, IDH status, and WHO grades of the two stemness subtypes in TCGA dataset. There were significantly more elderly patients (age >= 60) in the S2 group than in the S1 group (*p* < 0.0001; Figure 6A). Moreover, we found that the S1 group had more patients with IDH mutantions (*p* < 0.0001; Figure 6B). Furthermore, the S1 group had more patients with WHO grade II glioma and fewer patients with WHO IV glioma. However, the S2 group had more patients with WHO grade IV glioma and fewer patients with WHO II glioma (*p* < 0.0001; Figure 6C). More importantly, these clinical characteristics of the two stemness subtypes could also be validated in the CGGA dataset (Figures 6D–F).

Subsequently, we evaluated the prognostic predictive value of the stemness subtypes in different grades. Considering that almost all the WHO IV glioma patients belong to the S2 group, survival analyses were only performed in patients with WHO grade II and III glioma. Stratified survival analyses showed that patients in the S2 group have a better prognosis than patients in the S1 group in both WHO II and III glioma patients in TCGA database (Figure 6G) and CGGA database (Figure 6H).

### Immunological Microenvironment in Stemness Subtypes of Glioma

**Figure 7:**
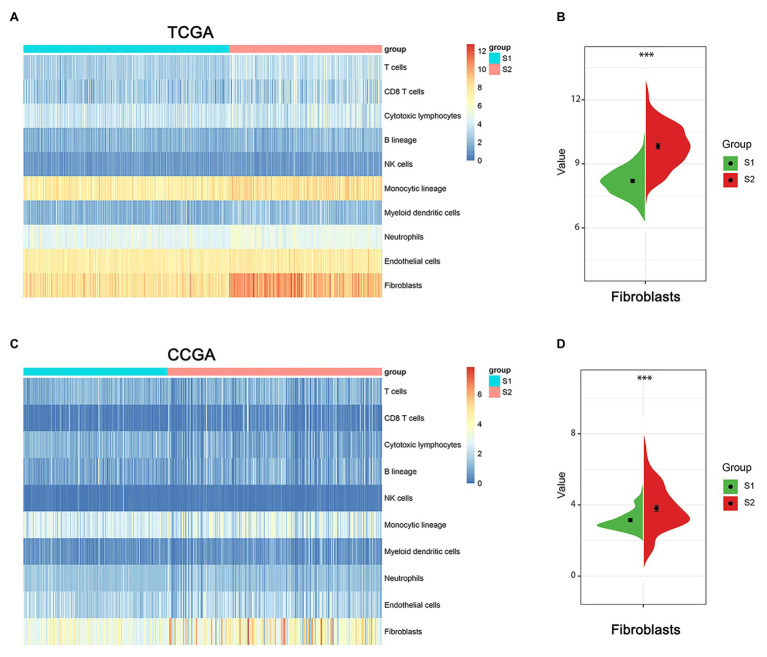
The immune cell infiltrate distribution of the two stemness subtypes. **(A)** Heatmap of immune infiltration cells in the two stemness subtypes of TCGA dataset. **(B)** The proportion of cancer-related fibroblasts in S1 and S2 group of TCGA dataset. ***represents *p* value of less than 0.001. **(C)** Heatmap of immune infiltration cells in the two stemness subtypes of CGGA dataset. **(D)** The proportion of cancer-related fibroblasts in S1 and S2 group of CCGA dataset. ***represents *p* value of less than 0.001.

The tumor microenvironment consists of the stromal and immune cells and plays a vital role in the aggressiveness of solid tumors. To measure the level of infiltrating immune cells in the tumor microenvironment, we also used MCP-counter estimates to examine the glioma samples in TCGA database. MCP analysis demonstrated that tumor-associated fibroblasts (CAFs) were significantly higher in the S2 group than in the S1 group (Figures 7A,B). Moreover, there was a similar finding in the CGGA dataset (Figures 7C,D).

### Functional Enrichment Analysis Between Two Stemness Subtypes

**Figure 8:**
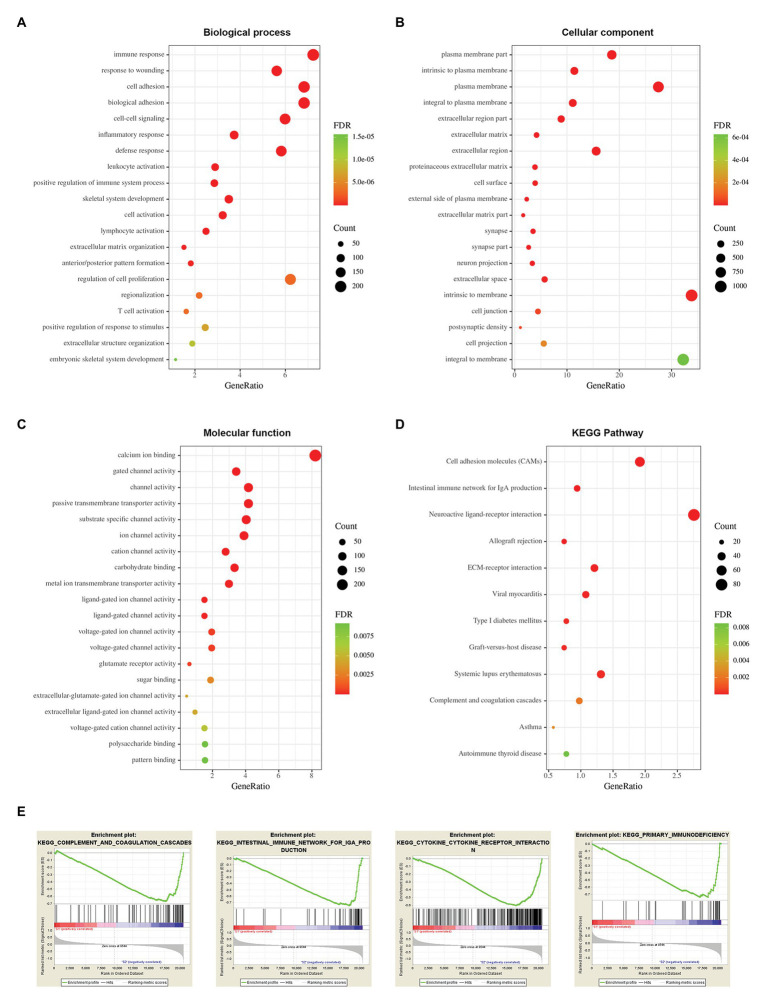
Functional enrichment analysis between two stemness subtypes of TCGA dataset. **(A)** The top 20 terms of biological processes (BP) in DEGs of two stemness subtypes. **(B)** The top 20 terms of cellular components (CC). **(C)** The top 20 terms of molecular functions (MF). **(D)** The Kyoto Encyclopedia of Genes and Genomes (KEGG) pathway in DEGs of two stemness subtypes. **(E)** The top four significantly enriched pathways of gene set enrichment analysis (GSEA).

To explore potential vital molecules and pathways contributing to different subtypes, we performed GO, KEGG, and GSEA analysis between the two stemness subtypes. Figures 8A–C shows the top 20 enriched GO terms in biological processes (BP), cellular components (CC), and molecular functions (MF). GO analysis revealed that immune response and cell adhesion were the main terms involved in BP; plasma membrane and extracellular matrix were significantly enriched in CC; calcium ion binding and channel activity were most enriched in MF (Figures 8A–C). The results of the KEGG pathway analysis showed that cell adhesion and immunological related pathways were mainly enriched (Figure 8D). GSEA showed the significantly enriched hallmark terms, including complement and coagulation cascades, cytokine receptor interaction, intestinal immune network for IgA production, and primary immunodeficiency (Figure 8E).

### Development and Validation of a Stemness Subtype-Related Prognostic Signature

Among the 3,129 candidate DEGs between the two stemness subtypes in TCGA dataset, 3,118 were identified as being independently associated with OS in univariate Cox regression analysis. The top 20 genes were used to perform multivariate Cox analysis. Based on the results of LASSO Cox regression analysis, a stemness subtype-related prognostic signature was developed. The risk score was calculated as follows: [Expression of PTRF × 0.08845 + Expression of ELF4 × 0.01153 + Expression of ELF5 × 0.2005 + Expression of BTN2A2 × 0.06099 + Expression of HMX1 × (−0.04178) + Expression level of FAAH × (−0.13481) + Expression of RGS16 × 0.10462 + Expression of IL4I1 × 0.12804 + Expression of LUZP2 × (−0.08583) + Expression of PLAT × 0.2138 + Expression of ETV2 × 0.15523].

**Figure 9:**
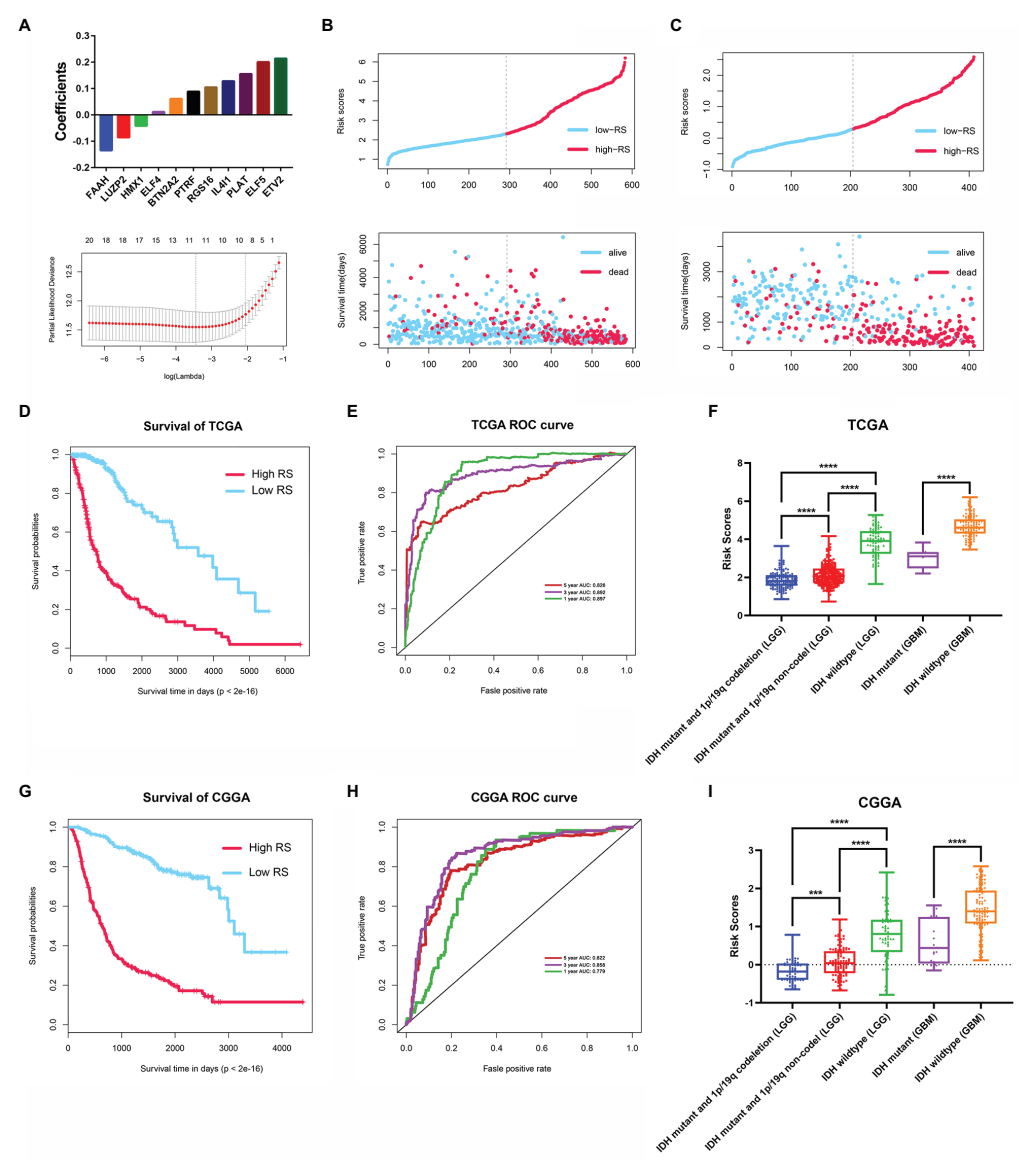
Construction and validation of the stemness subtype-related risk score model. **(A)** The 11 genes were selected by least absolute shrinkage and selection operator (LASSO) Cox analysis in TCGA dataset. Risk scores and living status for each patient in the training cohort **(B)** and the validation cohort **(C)**. **(D)** Kaplan-Meier curves of the OS of each patient in the training cohort. **(E)** Time-dependent ROC curve analysis of the risk score model in the training cohort (TCGA dataset). **(F)** The risk score distributions in different molecular subtypes of glioma (TCGA dataset). **(G)** Kaplan-Meier curves of the OS of each patient in the validation cohort (CCGA dataset). **(H)** Time-dependent ROC curve analysis of the risk score model in the validation cohort (CCGA dataset). **(I)** The risk score distributions in different molecular subtypes of glioma (CGGA dataset). ^***^p < 0.001 and ^****^p < 0.0001.

In this prognostic signature, eight genes were negatively associated with OS, and three were positively associated with OS (Figure 9A). Based on this prognostic signature, the risk score for each patient was calculated. According to the median cutoff value of risk scores, all patients were divided into high‐ and low-risk groups in both the training (TCGA dataset) and validation cohorts (CGGA dataset). The distribution of living status and time for each patient in the training and validation cohorts are shown in Figures 9B,C. Patients in the high-risk group had a shorter OS than patients in the low-risk group in the training cohort (*p* < 0.001; Figure 9D). The time-dependent ROC curve analysis showed that the AUC values of 1, 3, and 5 years were 0.897, 0.892, and 0.826 in the training cohort, respectively (*p* < 0.001; Figure 9E). Furthermore, glioma samples (both LGGs and GBM) with an IDH1-mutant type have lower risk scores than IDH1wild-type samples, and the risk scores in LGGs with IDH1-mutant and 1p/19q codeletion samples have lower risk scores than IDH1-mutant and 1p/19q non-codeletion samples (Figure 9F). Moreover, we assessed this risk score formula in the CGGA dataset and also found that patients with high-risk scores had poor prognosis in the validation cohort (*p* < 0.001; Figure 9G). The time-dependent ROC curve analysis showed that the AUC values of this risk score formula at 1, 3, and 5 years were 0.779, 0.858, and 0.822 in the validation cohort, respectively (Figure 9H). The risk scores were also consistent well with the molecular subtypes of glioma (Figure 9I).

### Development and Evaluation of the Nomogram

**Figure 10:**
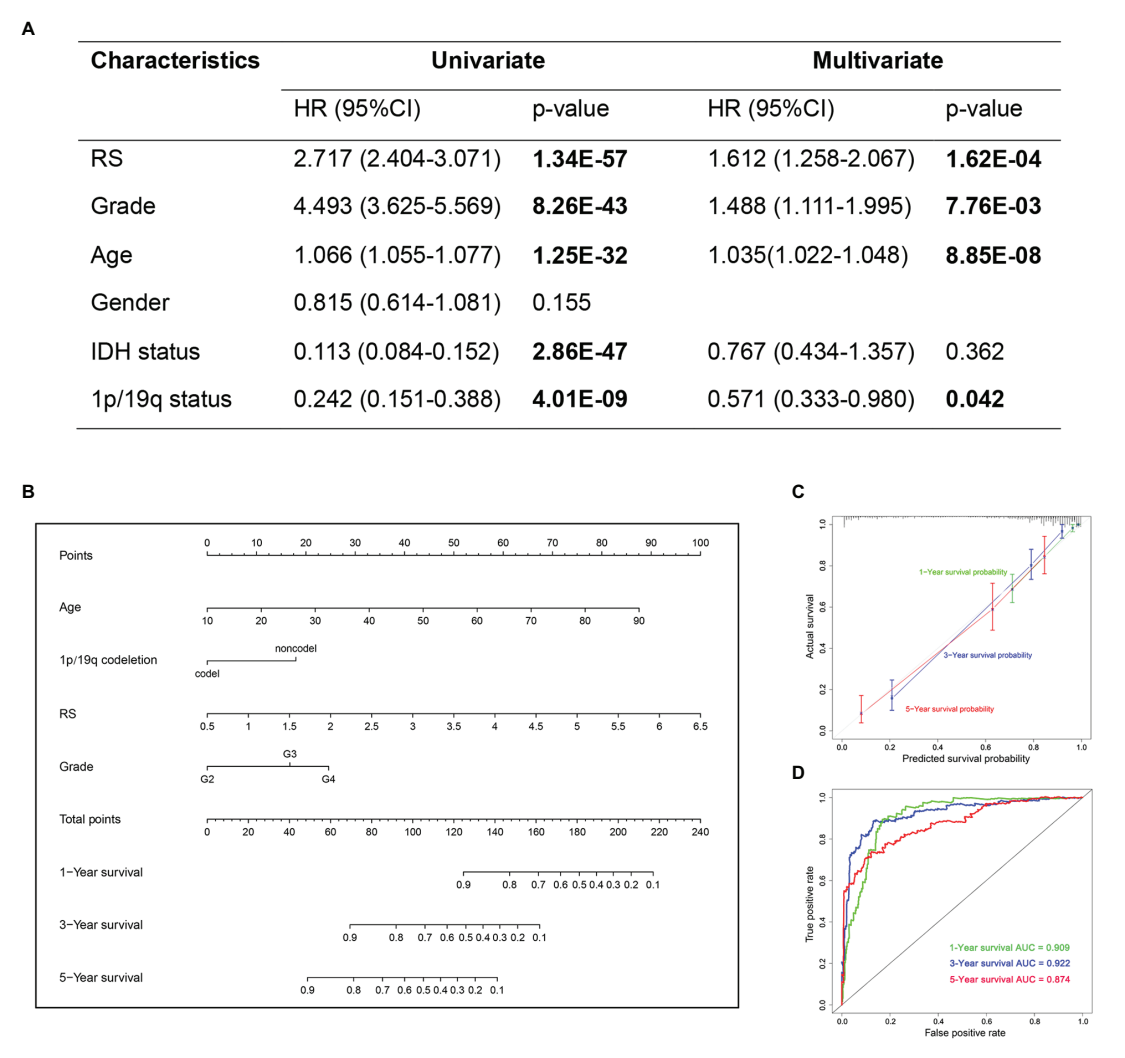
The nomogram based on TCGA dataset for survival prediction in glioma patients. **(A)** Univariate and multivariate Cox regression analysis of clinical features. **(B)** Development a nomogram for the quantitative prediction of 1-, 3-, and 5-years survival for LGG patients. **(C)** The calibration curves for predicting glioma patient 1-, 3-, 5-years survival. **(D)** The 1-, 3-, and 5-year time-dependent ROC curves of the nomogram.

In the univariate Cox analysis, the results showed that the risk score, grade, age, IDH status and 1p/19q status were significantly associated with OS. Then, we performed multivariate Cox regression analyses and the results showed that risk score (HR 1.612; 95% CI 1.258–2.067; *p* = 1.62E-04), grade (HR 1.488; 95% CI 1.111–1.995; *p* = 7.76E-03), age (HR 1.035; 95% CI 1.022–1.048; *p* = 8.85E-08), and 1p/19q status (HR 0.571; 95% CI 0.333–0.980; *p* = 0.042) were independently related to OS (Figure 10A). Based on the risk score and independent prognostic factors (grade, age, and 1p/19q status) in TCGA dataset, we constructed a nomogram model to predict the prognosis of glioma (Figure 10B). The calibration plot showed that the predicted values of OS at 1-, 3-, and 5-years for glioma patients had a good correlation with the actual values (Figure 10C). Then, the ROC curve analysis of the nomogram also showed a satisfactory evaluation for sensitivity and specificity with a 1-year AUC of 0.909, 3-years AUC of 0.922, and 5-year AUC of 0.874 (Figure 10D).

### ETV2 Is Involved in the Migration, Invasion, and EMT Process of Glioma Cells

**Figure 11:**
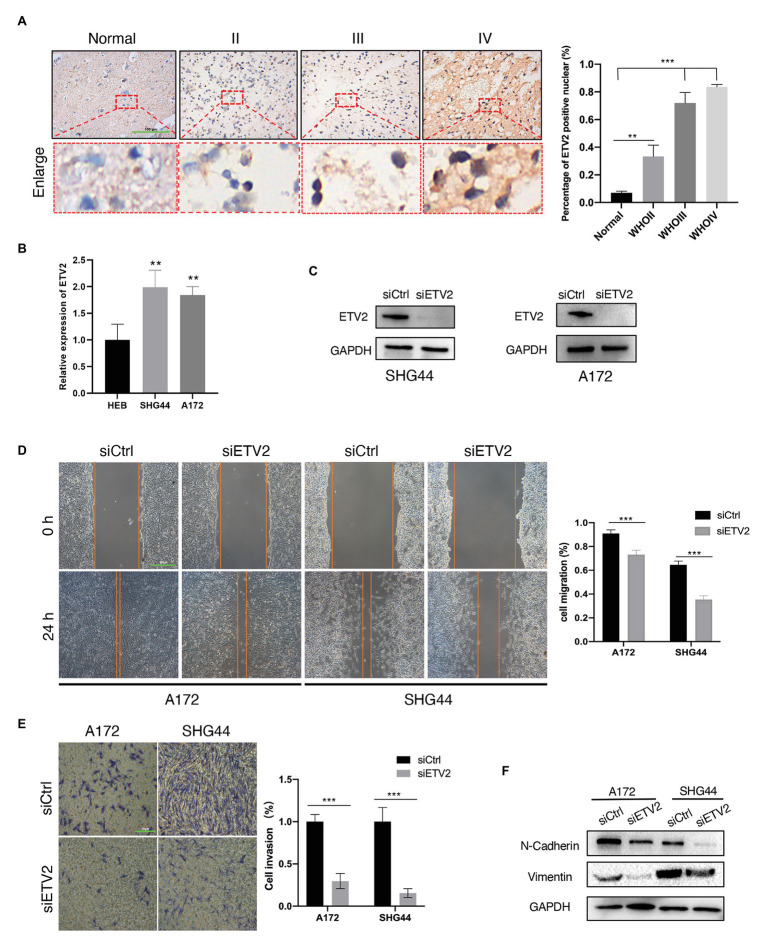
ETV2 is involved in the migration, invasion, and epithelial-mesenchymal transition (EMT) process of glioma cells. **(A)** Expression of ETV2 in IHC stained images of glioma tissues compared with adjacent non-tumor tissues was showed. For quantification, three para-cancerous tissues and five glioma sample for tumor groups were counted, mean values with SD are given, *p*-value is calculated by unpaired *t*-test, ^**^p < 0.01 and ^***^p < 0.001. **(B)** q-PCR showed the relative expression of ETV2 in HEB, SHG44, and A172 cells. **(C)** Western blot showed the ETV2 siRNA knockdown efficiency in SHG44 and A172 cells. **(D)** Wound healing assay showed that ETV2 knockdown led to profound impairment in cell migration ability of A172 and SHG44 relative to the control. **(E)** Invasion assays showed that ETV2 depletion resulted in suppression of A172 and SHG44 cell invasion capability. **(F)** Vimentin and N-cadherin levels were detected by western blot analysis in A172 and SHG44 cells after ETV2 knockdown with siRNA. For **(D,E)** mean values with SD form three independent experiments are given. *p*-value is calculated by unpaired *t*-test, ^***^p < 0.001.

Among the eight genes that were negatively associated with OS, we tested the expression of ETV2 in clinical tissue samples and glioma cell lines. First, we tested the expression of ETV2 in clinical tissue samples with immunohistochemistry. We found that the expression of ETV2 in clinical patients was correlated with WHO grade (Figure 11A). We also tested the expression of ETV2 in the SHG44 and A172 cell lines with q-PCR, and the expression of ETV2 in glioma cell lines (SHG44 and A172) was higher than that in the normal human cell line (HEB; Figure 11B). Next, we evaluated the effect of ETV2 on glioma cancer cell migration and invasion. Our results revealed that ETV2 knockdown dramatically impaired the cell migration ability of SHG44 and A172 cells relative to the control (Figures 11C,D). In *in vitro* invasion assays, the invasion potential was obviously suppressed due to the depletion of ETV2 (Figures 11E,F). These findings showed that ETV2 was a significant oncogene associated with the metastatic phenotypes of glioma cells.

EMT may promote the increased migratory capacity and invasiveness of tumor cells (Zhang et al., 2020b). Therefore, we investigated whether ETV2 mediates EMT in glioma. Considering the reduction in epithelial cells, E-cadherin was poorly expressed in glioma, so we tested the expression of vimentin and N-cadherin to analyze the EMT process in glioma (Liu et al., 2019). The expression of a mesenchymal marker, N-cadherin, decreased after ETV2 knockdown *via* siRNA. Vimentin is an intermediate filament protein that plays key roles in the integration of cytoskeletal functions and cellular migration. N-cadherin was also decreased after ETV2 knockdown both in both SHG44 and A172 cells. These results indicated that ETV2 is required for the EMT process of glioma.

## Discussion

Glioma is the most common and invasive primary brain tumor in adults. Tumor recurrence and treatment resistance are the obstacles to the treatment of glioma. CSCs play essential roles in these processes. Investigating the characteristics of CSCs may facilitate diagnosis, treatment, and prognostic prediction (Chai et al., 2018). In this study, we found that novel molecular subtypes, which based on the stemness index, could effectively predict prognosis in glioma patients. Moreover, the clinical characteristics (age, IDH status, and WHO grades) and tumor microenvironment of the two stemness subtypes are different. This typing could also be validated by the external dataset. Based on the DEGs of the two subtypes, we established a risk score model and a nomogram that could effectively predict the OS of glioma patients. Finally, we selected one gene (ETV2) from the risk score model for experimental validation. To our knowledge, this is the first study to provide a new type of glioma based on the mRNAsi-related genes.

Cancer stemness signatures, which are based on gene expression differences, have been applied to assess the clinical prognosis of some types of tumors (Ben-Porath et al., 2008; Eppert et al., 2011; Pinto et al., 2015). In 2018, Malta et al. (2018) proposed the conception of mRNAsi, which is considered as a more comprehensive index to uncover the characteristics of CSCs. Subsequently, mRNAsi was widely applied to reveal the stemness-related characteristics of different cancers, such as lung cancer (Qin et al., 2020; Zhang et al., 2020a), bladder cancer (Pan et al., 2019), endometrial carcinoma (Liu et al., 2020), medulloblastoma (Lian et al., 2019), and breast cancer (Pei et al., 2020). Moreover, mRNAsi was also used to identify the prognostic biomarkers and therapeutic targets associated with CSC characteristics of glioma (Lvu et al., 2020; Xia et al., 2020). Compared with a previous study, we performed the consensus clustering and identified novel molecular subtypes (S1 and S2 group) of glioma based on mRNAsi-related genes. We also constructed a nomogram model to predict the prognosis of glioma, which has potential for clinical application. More importantly, we performed some *in vitro* experiments to confirm the function of ETV2 in glioma cells.

Gliomas are the most frequent intrinsic tumors of the central nervous system. In the revised fourth edition of the WHO classification of CNS tumors published in 2016, the status of IDH and 1p/19q codeletion was applied in classification (Zhang et al., 2013). In our study, patients in subtype I (S1 group) were younger, more likely to have IDH-mutant status, lower WHO grades and poor prognosis than those in the subtype II (S2 group). Based on our risk score model, we found that glioma samples (both LGGs and GBM) with an IDH1-mutant type have lower risk scores than IDH1wild-type samples, and the risk scores in LGGs with IDH1-mutant and 1p/19q codeletion samples have lower risk scores compared with than IDH1-mutant and 1p/19q non-codeletion samples. The immunological microenvironments between the two subtypes were also different. Gliomas in subtype II (S2 group) are more likely to have a higher proportion of tumor-related immune and stromal cells, especially CAFs. CAFs, one of the main cellular components of the tumor microenvironment, play an important role in promoting cancer cell invasion and dissemination (Hurtado et al., 2020). These results indicated that this new typing could provide novel mechanistic and clinical insights for the diagnosis, treatment, and prognostic prediction of glioma patients.

Functional enrichment analysis of the DEGs between two stemness subtypes showed that immune and ECM-related GO terms and pathways were mainly enriched. This implied that the immunological microenvironment and ECM components might have a close relationship with the stemness characteristics of glioma. Previous studies have shown that glioma stem cells interact with immune cells and simulate the early microenvironment during tumorigenesis (Zhai et al., 2020). CSCs can protect cancer cells from immune attack by producing immune inhibitory factors to communicate with tumor microenvironment components (Khosravi et al., 2020). Moreover, CSCs contribute to glioma invasiveness, which is closely correlated with the extracellular matrix (Ortensi et al., 2013). These findings were in accordance with our findings.

In the present study, we also developed a stemness subtype-related prognostic signature. There were 11 essential prognostic genes in this signature, which have not been reported in previous glioma stemness index related publications (Lvu et al., 2020; Xia et al., 2020). Our results are a good supplement to the existing research about the prognosis prediction of patients with glioma. Among the three prognostic genes that were positively associated with OS, LUZP2 is a protein limitedly expressed in the brain and spinal cord. A recent study showed that low LUZP2 expression independently predicted poor OS in LGG (Li et al., 2020). Fatty-acid amide hydrolase (FAAH), an intracellular serine hydrolase, plays an important role in the inhibition of stem cell migration (Wollank et al., 2015). Among the eight genes that were negatively associated with OS, ETV2, ELF5, IL4I1, and BTN2A2 were novel prognostic biomarkers for glioma, which have not yet been reported yet. ETV2 is a critical factor for vascular development and regeneration, which may contribute to tumorigenesis (Baltrunaite et al., 2017; Choi, 2018). In GBM, ETV2 is sufficient and necessary for the trans-differentiation of GBM stem cells to an endothelial lineage (Humm and Sylvia, 1965). In our study, we found that ETV2 is negatively associated with OS. The expression of ETV2 is closely associated with WHO grade. More importantly, *in vitro* experiment revealed that ETV2 is involved in the migration, invasion, and EMT process of glioma cells. ELF5 is an epithelial-specific member of the E26 transforming sequence (ETS) transcription factor family, which plays critical roles in malignancy, particularly in basal-like and endocrine-resistant forms of breast cancer (Piggin et al., 2016; Singh et al., 2020). IL4I1 and BTN2A2 are both involved in the regulation of the immunologic microenvironment in different tumors (Smith et al., 2010; Sarter et al., 2016; Molinier-Frenkel et al., 2019; Sadik et al., 2020). However, what is their roles in tumorigenesis and progression of glioma are still need further investigation.

There were some limitations in our study. First, there were only very limited normal samples (only five normal samples) included in our study, which might lead to there being no difference in mRNAsi between normal and tumor samples. Second, DEG analysis might neglect some potential mRNAs that were closely related to the mRNAsi. Third, this study is a retrospective study, and the stemness-related typing of glioma should be further confirmed by prospective studies. Finally, the underlying mechanisms of the selected genes in the model affecting the prognosis of glioma should be elucidated by more experiments *in vivo*.

In this study, we identified two distinct stemness-related molecular subtypes of glioma, which could provide new insights for the development of precision diagnosis and prognostic prediction for glioma patients. Moreover, we developed a stemness subtype-related prognostic signature that could effectively predict the prognosis of glioma patients.

## Data Availability Statement

The original contributions presented in the study are included in the article/supplementary material, further inquiries can be directed to the corresponding authors.

## Author Contributions

XJ and WY conceived and designed the study. JT and WY wrote the manuscript. YC, ZX, QZ, and CZ analyzed the results. GT, HL, SW, YG, and ZJ performed the image visualization. JT, HZ, MZ, and CR performed the validation experiment. All authors contributed to the article and approved the submitted version.

### Conflict of Interest

The authors declare that the research was conducted in the absence of any commercial or financial relationships that could be construed as a potential conflict of interest.

## Footnotes

^1^https://xena.ucsc.edu/public

^2^http://www.cgga.org.cn/index.jsp

^3^https://string-db.org/

^4^http://software.broadinstitute.org/gsea/msigdb/
